# Advances in Cider Flavor: Integrating Apple Raw Materials, Microbial Ecology, and Process Control

**DOI:** 10.3390/microorganisms14081802

**Published:** 2026-08-15

**Authors:** Zhiyong Zhang, Chan Yan, Junjie Li, Lina Zhao, Lang Li, Rongjing Cai, Wenhui Wei, Shaokun Lu

**Affiliations:** 1 School of Chemistry and Chemical Engineering, Zhaotong University, Zhaotong 657000, China; 18183861697@163.com (C.Y.); lijunjie900501@163.com (J.L.); 15733200328@163.com (L.Z.); llhwls@163.com (L.L.); 2 School of Agriculture and Life Sciences, Zhaotong University, Zhaotong 657000, China; 20240001@ztu.edu.cn; 3 College of Big Data, Yunnan Agricultural University, Kunming 650201, China; lsk999@126.com

**Keywords:** cider, flavor, apple cultivar, non-*Saccharomyces*, lactic acid bacteria, oxygen, nutrient

## Abstract

Cider is a low-alcohol fruit wine produced by partial or complete fermentation of apple juice. In recent years, its consumption has grown significantly, especially in Asia. Its distinctive flavor profile reflects a complex interplay of aroma and non-volatile components. The composition of cider is mainly influenced by three factors: raw materials, starter cultures, and production process. This review begins by outlining the composition of cider, covering both its aromatic compounds and non-volatile components. The primary aroma profile is defined by higher alcohols, esters, fatty acids, and carbonyl compounds, while sugars, organic acids, and polyphenols are the key determinants of its taste. Subsequently, it provides a detailed analysis of how the raw material, the choice of starter cultures, and the applied production processes collectively shape the cider’s flavor. Research shows that apple variety and maturity influence the levels of sugars, organic acids, and polyphenols, shaping the flavor foundation of cider. To enhance flavor diversity, inoculation strategies have shifted from single-strain fermentation with *Saccharomyces cerevisiae* to mixed fermentations using non-*Saccharomyces* yeasts and lactic acid bacteria, either simultaneously or sequentially. Currently, screening non-*Saccharomyces* strains has become a key strategy to increase cider flavor complexity. Precise micro-oxygenation and nutrient supply regulate microbial metabolism, thereby controlling the fermentation process and the production of specific flavor compounds. In the future, given the untapped diversity of non-*Saccharomyces* yeasts and lactic acid bacteria in winemaking traits, research in this direction may be key to improving cider quality. In addition, selecting apple cultivars tailored to specific cider styles and exploring pre-fermentation treatments such as cold maceration and enzymolysis may also contribute to enhancing the flavor diversity of cider.

## 1. Introduction

Cider is the world’s second-largest fruit wine category [1]. The term cider refers to artisanal products made by the partial or complete fermentation of apple juice, including flavored variants that incorporate other fruits [2]. Cider has a relatively low alcohol content, ranging from 1.2% to 8.5% (*v*/*v*). It is positioned by the market as a natural and refreshing beverage [3]. European countries such as the United Kingdom, Ireland, France, and Spain have long-standing traditions of cider production, with different regions developing unique brewing styles [4]. Globally, fermented apple beverages are known by various names, such as cider, cidre, sidre, sidra, and apfelwein (Table 1). However, in North America, the term “cider” typically refers to cloudy, unfiltered, and unpasteurized apple juice, while the fermented alcoholic product is specifically called “hard cider” [5]. Although cider can be produced from any apple cultivar, it is typically made from specific varieties with higher sugar and phenolic contents [6]. Furthermore, depending on the region of production, cider may be made exclusively from apples or may include the addition of pears or concentrated apple juice. Additives such as sugar, acid, or colorants may also be used [5]. Based on production techniques and cultural traditions, ciders are generally categorized into two main types: Standard Ciders (including New World Cider, English Cider, French Cider, etc.) and Specialty Ciders (such as New England Cider, Flavoured Cider, Ice Cider, etc.) [7]. “Ice Cider,” a relatively recent style, originated in Quebec, Canada. It is produced using apples that have naturally frozen on the tree, resulting in a must with a high sugar concentration [8].

**Figure 1:**
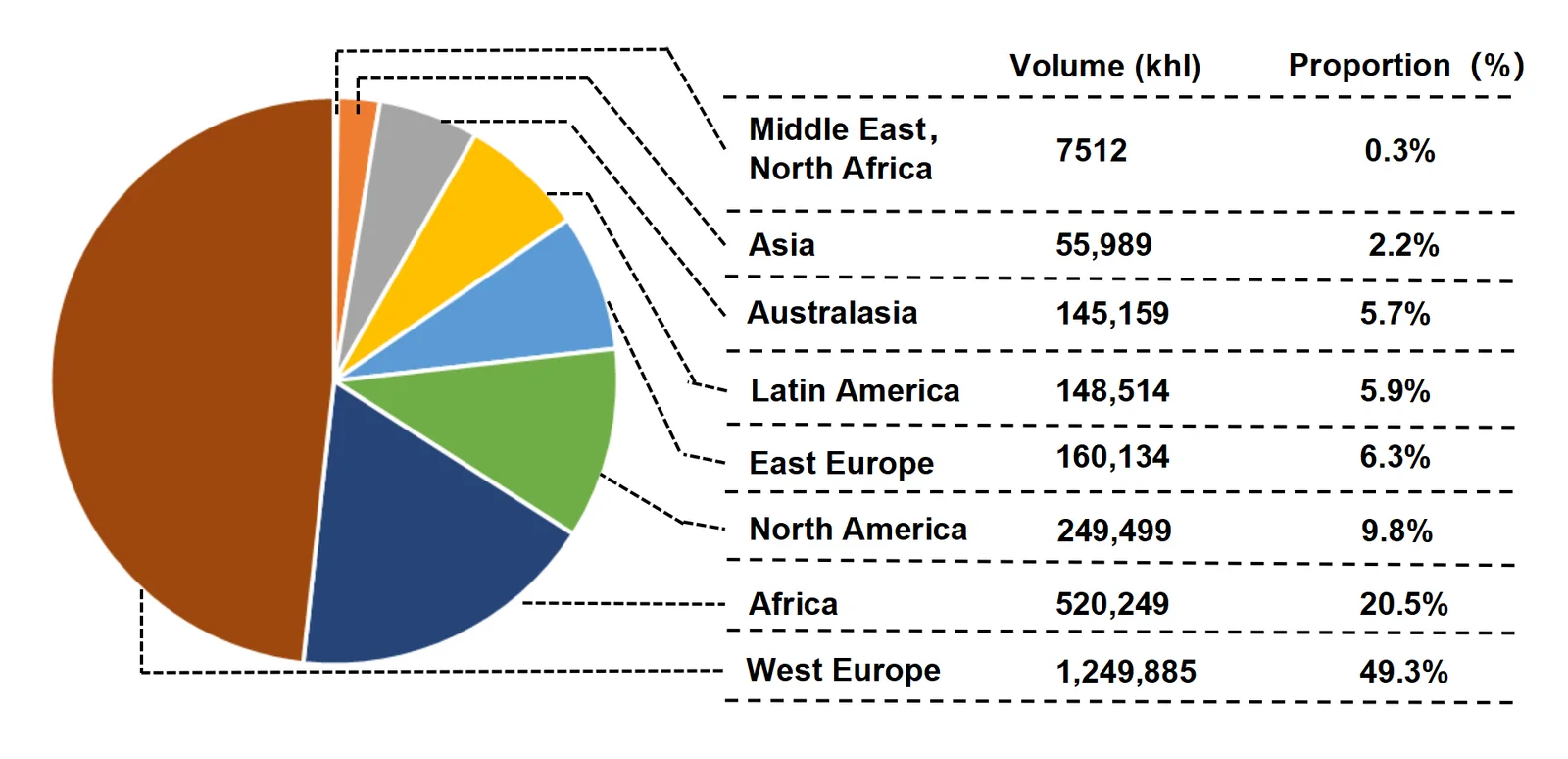
Regional share of consumption in 2024. Data from AICV’s official website: https://aicv.org/en.

Cider is experiencing a surge in both consumption and production globally. The global cider market is valued at 26 million hectoliters, with a relatively modest compound annual growth rate (CAGR) of 0.8% over the past five years. As shown in Figure 1, the consumption volume of cider across different regions in 2024 highlights Western Europe’s dominant share of 49.3%, followed by Africa at 20.5%, while Asia contributes only 2.2% (source: https://aicv.org/en). In recent years, Western Europe has seen a slight decline in consumption, whereas emerging markets in Asia, Africa, Eastern Europe, and Latin America have become the primary drivers of growth—with Asia being the fastest-growing region. According to statistics from the Food and Agriculture Organization of the United Nations (FAO), China ranks first globally in both apple cultivation area and production output, accounting for over 50% of the world’s total (source: https://www.fao.org/faostat/en/#data (accessed on 1 June 2026)). Looking forward, as consumer habits evolve and acceptance of cider grows, the Asian cider market holds considerable potential for innovation and vigorous development.

Despite similarities between apple wine fermentation and grape wine making, there are significant differences in apple juice composition, such as higher fructose and L-malic acid content and lower yeast assimilable nitrogen [9,10]. These factors affect microbial activity differently compared to grape must, ultimately leading to distinct compositional and sensory profiles (taste and odor) in cider versus wine. Current research mainly focuses on how apple variety, starter culture, and fermentation technology affect the sensory quality and functional properties of cider. Among these, the screening of non-*Saccharomyces* yeast strains is a research hotspot. Fabien J. Cousin et al. summarized the microbial communities in cider and their impact on sensory quality [3]. Paul Cristian Calugar et al. reviewed the effects of apple varieties and microorganisms on the volatile composition of cider [11]. However, with the rapid growth of cider research, new findings have continuously emerged, including non-*Saccharomyces* yeast strains (e.g., *Kluyveromyces marxianus*), mixed-culture inoculation strategies (such as simultaneous inoculation of non-*Saccharomyces* yeast and lactic acid bacteria), and micro-oxygenation fermentation technology, among others. Therefore, it is necessary to update the review of recent findings, especially regarding the fermentation characteristics of non-*Saccharomyces* yeasts in cider making.

This review first outlines the flavor basis of cider (non-volatile and aroma components) and the anabolic pathways of related metabolites. It then summarizes the effects of apple raw materials and starter cultures (non-*Saccharomyces* yeasts and lactic acid bacteria) on cider flavor. Furthermore, it presents research advances in regulating microbial metabolism by controlling oxygen and nutrient supply, which ultimately impacts cider quality.

## 2. Ingredients of Cider: The Foundational Basis of Flavor

The composition of cider is primarily determined by that of the apple juice. Therefore, the apple variety, cultivation conditions, fruit maturity, as well as physical damage (such as cracks and bruises) or biological damage (e.g., mold, insect infestation) all influence the final composition of the cider [12]. Secondly, it depends on the fermentation process and the activity of microorganisms during fermentation [13]. The main constituents of cider include water, carbohydrates, organic acids, polyphenols, and volatile aroma compounds [14].

### 2.1. Volatile Components

The aroma of fruit wine is primarily derived from three sources: varietal aroma, which refers to the volatile compounds inherent to the raw material; fermentation aroma, comprising secondary products formed during fermentation; and post-fermentation aroma, which consists of unique mixtures developed during the aging process [15,16]. The raw material variety can largely define the wine’s aroma profile, especially in young fruit wines. Aromatic compounds in fruit may exist in free forms that directly contribute to scent, but more frequently, they are present as non-volatile, sugar-bound precursors. Odorless glycosides serve as these aroma precursors, accumulating during fruit ripening and are predominantly located in the peel. Glycosides release odor-active aglycones through acid or enzymatic hydrolysis (by plant, yeast, or added β-glucosidase). When the levels of these aglycones exceed their perception thresholds, they become the active aromatic components. Volatile compounds synthesized by fruit wine yeast during fermentation mainly include higher alcohols (imparting fusel, almond, and floral notes), medium- and long-chain volatile acids (contributing fatty, cheesy, or sweaty odors), acetate and ethyl esters (providing fruity and floral aromas), and aldehydes (imparting buttery, fruity, or nutty notes). Furthermore, aromatic substances can interact with each other or with other molecules in the wine matrix—such as oxygen, proteins, polyphenols, and polysaccharides—thereby altering their sensory impact [17].

Although some aroma compounds in cider are already present in the apple juice (e.g., hexanol, ethyl 2-hydroxyhexanoate, ethyl lactate), the majority are generated during fermentation. The most significant aroma compounds in apple cider are higher alcohols, esters, fatty acids, carbonyl compounds (aldehydes and ketones), and terpenes. Table 2 lists the types, concentrations, and odor characteristics of the major aroma compounds in cider. The content of these aroma compounds varies considerably across different ciders due to differences in apple cultivars, fermentation microorganisms, and brewing techniques.

**Figure 2:**
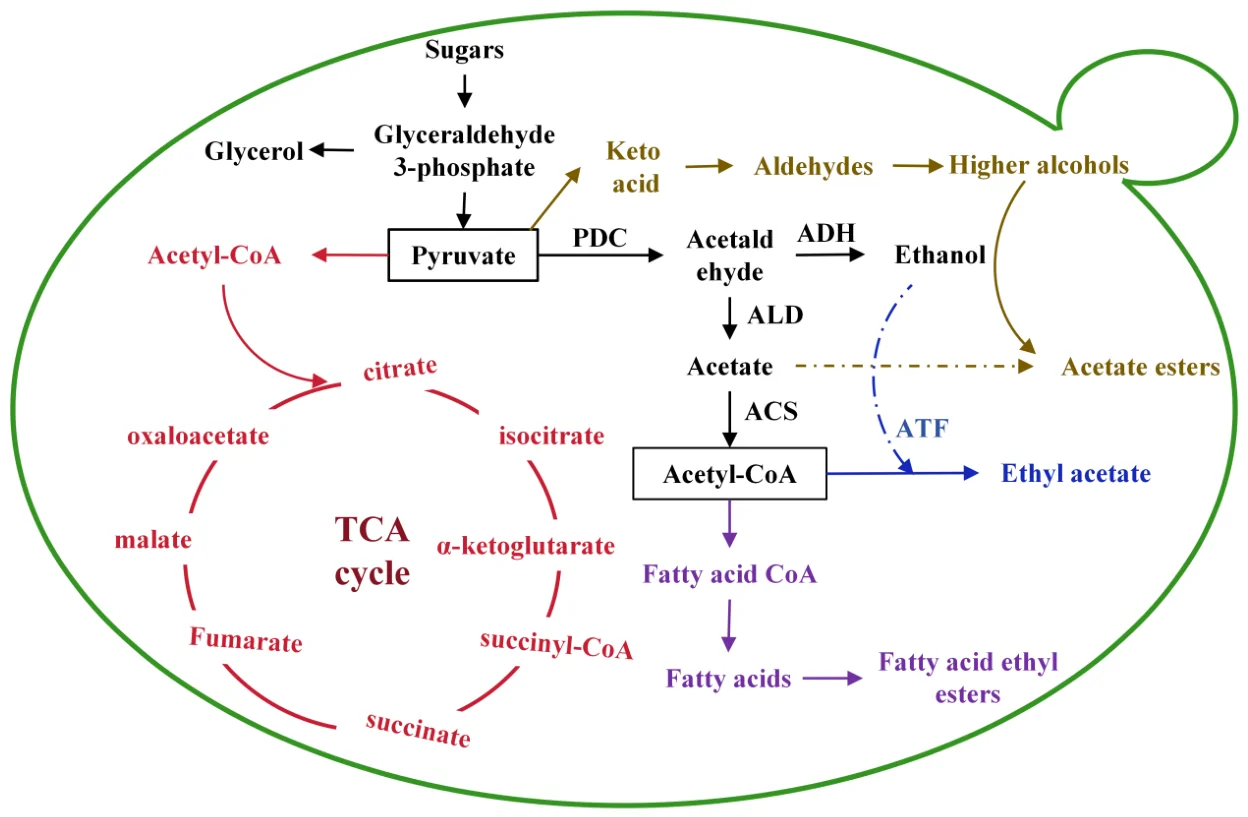
Overview of the central carbon metabolism and the synthesis of volatile and non-volatile compounds in yeast. PDC, pyruvate decarboxylase; ADH, alcohol dehydrogenase; ALD, acetaldehyde dehydrogenase; ACS, acetyl-CoA synthetase; ATF, alcohol acetyltransferase. Different colors indicate the metabolic pathways of different aroma compounds. References: [18,22,26].

The synthesis of volatile compounds in yeasts are showed in Figure 2. Higher alcohols refer to alcohols containing more than two carbon atoms. Most higher alcohols are produced by yeast through the biosynthetic pathway of sugar metabolism or the catabolic Ehrlich pathway of amino acids, with the formation of intermediate keto acids being a vital step in both [24]. In media with low yeast-assimilable nitrogen, higher alcohols are primarily formed via the biosynthetic pathway rather than through amino acid catabolism [18]. The predominant higher alcohols in apple cider are 2-phenylethanol and 3-methyl-1-butanol (isoamyl alcohol) [12,19]. Higher alcohols serve as precursors to esters and positively contribute to the aromatic complexity of fruit wine. However, at concentrations exceeding 300 mg/L, they can produce intense, pungent odors. During fruit wine maturation, higher alcohols can also be oxidized into aldehydes. The apple raw material is the primary source of aldehydes; after microbial fermentation, both the variety and concentration of aldehydes decrease [25].

Fatty acids, particularly acetic, hexanoic, octanoic, and decanoic acids, help create a fresh aroma in the wine. Esters are primarily classified into two groups based on their formation: acetate esters and ethyl esters. They are synthesized via esterification, with acetate esters resulting from acetic acid and higher alcohols, and ethyl esters from ethanol and medium-chain fatty acids [20]. In cider, esters constitute nearly 50% of the total variety of volatile compounds [21]. Esters with odor activity values (OAVs) above their thresholds primarily include acetate esters (e.g., ethyl acetate, isoamyl acetate, hexyl acetate) and ethyl esters of medium-chain fatty acids (e.g., ethyl hexanoate, ethyl octanoate, ethyl decanoate) [27]. In young wine, ethyl acetate contributes significantly to the fruity aroma, but high concentrations can suppress fruity and floral notes [28]. Esters at appropriate concentrations impart a pleasant fruity character to the wine, whereas excessive levels can introduce undesirable solvent-like or nail polish off-odors [17].

Semi-volatile organic compounds (SVOCs) are increasingly recognized for their contribution to beverage aroma. Characterized by boiling points between 200 and 400 °C, these compounds can exist in both gaseous and particulate phases at room temperature [29]. Ciro Orecchio et al. characterized SVOCs in 56 Italian ciders using comprehensive two-dimensional gas chromatography coupled with time-of-flight mass spectrometry (GC×GC-TOF-MS). The detected SVOCs, with an average molecular weight of around 160 Da, encompassed alcohols, cyclic hydrocarbons, esters, and terpenes [30]. However, research focusing on SVOCs in cider remains relatively limited.

To date, hundreds of volatile compounds have been identified in cider using headspace solid-phase microextraction (HS-SPME) combined with GC-MS. However, the concentrations of these aroma compounds are generally low (particularly for esters, often at the μg/L level) and are markedly influenced by factors such as raw materials, yeast strains, and fermentation conditions. Moreover, most studies rely on internal standard methods for semi-quantitative analysis of aroma components. These factors make it difficult to directly compare the reported concentrations of the same aroma compound across different studies.

### 2.2. Non-Volatile Components

Sugars, organic acids, and polyphenols significantly contribute to the taste of cider. They interact to create an integrated sensation in the mouth. Sugars contribute to perceived sweetness, with fructose and sorbitol largely responsible for the sweet flavor of the final product. Organic acids define the cider’s taste profile and directly influence the perception of sweetness; a positive perception of acidity is associated with the product’s freshness. Polyphenols primarily influence the bitterness, astringency, and color of the product [5]. Polyphenols like epicatechin, catechin, and procyanidins (trimers C1, dimers B1, B2) contribute to the bitter taste of cider, while phenolic acids such as coumaric, gallic, and protocatechuic acids are responsible for its astringency [31]. Proanthocyanidins significantly contribute to the astringent mouthfeel of the wine, and a higher degree of polymerization leads to increased bitterness and astringency in the wine body [14].

The sugar content in cider is as follows: fructose (3.0–32.9 g/L), sorbitol (3.1–14.1 g/L), glucose (0.1–20.8 g/L), sucrose (0.1–11.8 g/L). The organic acid content is: malic acid (0.37–2.9 g/L), citric acid (0.0–2.8 g/L), L-lactic acid (0.04–4.35 g/L [20,21]. Generally, cider contains fewer phenolic compounds than the corresponding apple juice. Apple juice contains more flavonoid glycosides and dihydrochalcones, while the fermentation process increases the content of free hydroxycinnamic acids in cider [32]. The phenolic compounds in apple cider fall into five categories: (1) Flavan-3-ols, including (+)-catechin, (−)-epicatechin, and their polymers, with a content range of 2–5 g/kg; (2) Hydroxycinnamic acids, including caffeic acid, p-coumaric acid, and ferulic acid, and their esters (e.g., chlorogenic acid, 5-caffeoylquinic acid), with a content range of 0.3–2.5 g/kg; (3) Dihydrochalcones, including phloridzin, phloretin, and phloretin-2′-xyloglucoside, with a content range of 0.02–0.10 g/kg; (4) Flavonols (e.g., quercetin, isorhamnetin); (5) Trace amounts of anthocyanins [5,32]. Flavonoids (114.81–253.44 mg/L) were the second most abundant class of phenolic compounds in ciders, following hydroxycinnamic acids (141.47–327.44 mg/L) [4]. Catechin (8.13 mg/L), epicatechin (24.44 mg/L), caffeic acid (20.72 mg/L), and chlorogenic acid (14.43 mg/L) are the major monomeric phenolic compounds in cider [33].

Compared with volatile components, research on non-volatile components in cider remains relatively limited. Current studies commonly employ untargeted metabolomics for measurement, with compound identification mainly relying on existing databases, while subsequent quantitative validation is insufficient. This leads to a limited discovery of novel functional compounds and compromised data reliability. Furthermore, the types of compounds subjected to quantitative analysis are mostly confined to a few amino acids, organic acids, and phenolic substances.

## 3. Factors Influencing the Flavor Profile of Cider

### 3.1. Apple Raw Materials: Cultivar and Maturity

Based on their malic acid and tannin content, apples can be classified into four types: sweet, bitter-sweet, bitter-sharp, and sharp. Fuji is the world’s most widely cultivated apple cultivar. In the European market, Golden Delicious, Jonagold, and Red Delicious collectively represent over 70% of the market share [12]. The raw materials for cider production primarily include fresh apples, dehydrated apples, apple juice (pulp), and concentrated apple juice. Generally, cider producers prefer specific apple varieties over dessert apples to produce high-quality ciders with a balanced profile of acidity, sweetness, astringency, and bitterness. In China, due to the lack of specialized cider apple varieties, fresh dessert apple varieties are commonly used in cider processing [34]. Ciders made from dessert apple varieties may exhibit more desirable mouthfeel and aroma, whereas those produced from varieties richer in polyphenols are often perceived as more astringent [35]. Consequently, the choice of apple cultivar serves as an essential strategy for enhancing cider quality.

Apple juice consists primarily of water (80–90%) but also contains various soluble compounds. Sugars constitute the main soluble components (approximately 75–90%) and exist in multiple forms: monosaccharides, oligosaccharides, and polysaccharides (e.g., amylose, amylopectin, pectin). Fructose is the predominant sugar. Malic acid (1–10 g/L) is the predominant organic acid in apple juice, constituting approximately 85% of the total acidity and, together with citric and quinic acids, accounting for 95.57% of the total organic acid content [6,13]. Amino acids are the primary nitrogen source for yeasts and also act as precursors for key volatile compounds, particularly higher alcohols and esters. Aspartic acid, glutamic acid, asparagine, and serine collectively account for over 80% of the total amino acids present. A deficiency of amino acids in apple juice can lead to sluggish fermentation and reduced aroma production. Furthermore, stuck fermentation is likely if the assimilable nitrogen concentration falls below 75 mg/L [36].

Different apple varieties exhibit significant differences in the content of organic acids, polyphenols, phosphates, minerals, and trace elements [37]. The main constituents of apple polyphenols include chlorogenic acid, phloridzin, proanthocyanidins, epicatechin, anthocyanins, and flavonoids. A strong correlation exists between apple polyphenols and their health benefits, such as anticancer, anti-inflammatory, antioxidant, and antimicrobial activities [38]. The types and concentrations of apple polyphenols vary depending on the cultivar and the growth stage [39]. Cider made from typical dessert apples (Red Delicious) contains higher levels of phenolic compounds than cider produced from varieties such as Pink Lady and Royal Gala [40]. Although the use of different yeast strains can influence the final astringency and bitterness of cider to some extent, the primary sensory differences originate from the apple variety itself [41].

The maturity of the apple raw material substantially impacts its compositional profile. As maturity increases, the sugar content of the fruit rises while acidity decreases [28]. The soluble solids content in apples of different maturity stages ranges from 8.0 to 18.9 °Brix, and malic acid content varies between 0.13 and 17.2 g/L [42]. Regarding phenolic compounds, unripe apples contain higher levels than ripe or overripe apples; ciders produced from them follow the same trend. Apple maturity also influences the composition of volatile compounds. Cider made from overripe apples contains more volatile compounds, such as higher alcohols and acetate esters, than cider from less ripe fruit [43]. Analysis of volatile compounds in apples at different maturity stages (unripe, ripe, and senescent) revealed that ripe apples possess the highest content of aroma substances [42].The phenolic content also varies markedly among different apple cultivars [44]. The apple variety further influences the composition and content of polyphenols in the corresponding pomace. The primary phenolic compounds in fresh apple pomace include chlorogenic acid, caffeic acid, (+)-catechin, (-)-epicatechin, rutin, and quercetin glycosides, among others [45].

Although a wide range of commercial apple varieties are available, there is currently no industry consensus on which varieties are suitable for cider making. Many dessert and wild varieties are also frequently used for cider fermentation. Moreover, the processing of apple raw materials lacks standardized practices: fresh juice and concentrate are all employed for fermentation, and whether to retain the peels and seeds remains unclear.

### 3.2. Starter Cultures: From Spontaneous Fermentation to Synthetic Microbial Community

Fermentation of fruit wines is primarily driven by microorganisms originating from the raw fruit materials, the winery environment and equipment, or by inoculated cultures. Yeast plays a decisive role in cider fermentation. The biosynthesis of yeast metabolites strongly influences the flavor complexity and mouthfeel of cider [46]. Traditional cider is produced through spontaneous fermentation by indigenous yeasts. *Saccharomyces cerevisiae* is typically absent or present at very low levels in unfermented juice yet rapidly dominates spontaneous fermentation to complete alcoholic conversion [47]. Cider production in regions such as France, Ireland, and Spain relies entirely on natural microbiota, requiring at least six weeks of fermentation before bottling. However, the population of wild microorganisms (e.g., bacteria and molds) on the peel and stems of ripe fruit far exceeds that of indigenous yeasts. These wild microbes frequently interfere with yeast fermentation, adversely affecting wine quality [20].

To accelerate fermentation and minimize sensory deviations in the product, starter cultures are often inoculated into apple juice [48]. *S. cerevisiae* is considered the traditional commercial yeast for cider production due to its robust fermentative capacity, high tolerance to harsh environments, and predictable production of flavor compounds [49]. On intact, ripe grapes, *S. cerevisiae* constituted only a minor part of the microbial flora. Other yeast species were far more numerous and play a crucial role in the initial hours of wine fermentation. In the middle and late stages of fermentation, *S. bayanus* and *S. cerevisiae* together accounted for over 50% of the isolates [50]. Analysis of the dynamic changes in the yeast community during spontaneous cider fermentation revealed that *Hanseniaspora* spp. and *Metschnikowia pulcherrima* thrive in the early stages. *S. bayanus* dominates the mid-fermentation phase. In contrast, *S. cerevisiae* became the predominant yeast in the late fermentation stage.

In fruit wine production, inoculating starter cultures—primarily yeasts and lactic acid bacteria—not only helps control fermentation but also improves flavor by reducing off-flavors from volatile acids, sulfur compounds, and phenolic compounds [37]. Table 3 lists yeast and bacterial strains used in cider fermentation. Inoculation alters the cider’s microbial composition. It suppresses other yeasts but increases bacterial diversity, making inoculated cider more bacteriologically diverse than spontaneously fermented cider [51]. In spontaneously fermented ciders, dominant bacteria included *Leuconostoc* genus, *Gluconobacter* genus, and *Rahnella* genus. Yeasts were primarily *Saccharomyces cerevisiae* (77.4%), alongside *Hanseniaspora* genus and *Torulaspora* genus. In contrast, inoculated fermentation largely suppressed *Firmicutes* [51]. Different yeast strains mainly affected anthocyanin and flavonoid profiles [52]. Compared to *S. cerevisiae*, *S. bayanus* produced ciders with higher levels of hydroxycinnamic acid derivatives [44]. *S. bayanus* var. *uvarum* exhibits a shorter lag phase, higher cell viability, and better cold tolerance than *S. cerevisiae* [53].

Commercial yeast has been widely used as a single inoculum in alcoholic fermentation [59]. However, fermentation relying solely on *S. cerevisiae* often results in a limited aroma profile, constraining the overall sensory complexity of the cider. Mixed-culture fermentations have been widely investigated as a strategy to address the limitations of single-strain inoculation. These desired sensory characteristics can be achieved when multiple yeasts participate in fermentation, as in spontaneous or mixed fermentations [60]. Non-*Saccharomyces* yeasts are naturally predominant in freshly pressed juice but were historically considered spoilage microorganisms. Current research has demonstrated that non-*Saccharomyces* yeasts (such as *Torulaspora delbrueckii*, *Pichia kluyveri*, *Hanseniaspora uvarum*, *Wickerhamomyces anomalus*, and *Metschnikowia pulcherrima*) possess excellent oenological properties for fruit wine fermentation [61,62]. These include the release of flavor and aroma compounds that positively contribute to the final wine’s sensory complexity [63]. The central carbon metabolism and the synthesis of main flavor compounds in yeast are showed in Figure 2. Acetyl-CoA is a key intermediate metabolite in the central carbon metabolic pathway of yeasts. The variation in aroma compounds production among different yeast strains during fermentation primarily arises from differences in their enzyme systems. Microorganisms synthesize key flavorzyme, which generate aroma compounds via proteolysis, lipolysis, and related reactions. *Metschnikowia koreensis* showed notable protease and lipase activities, which contributed to its fermentation performance [64]. Yeasts from the genera *Hanseniaspora* and *Torulaspora* were used to enhance the aroma and quality of wine. In *Hanseniaspora*, the absence of genes for branched-chain amino acid transaminases and acyl-CoA/ethanol O-acyltransferases reduces the production of branched-chain higher alcohols, fatty acids, and ethyl esters [47]. *Torulaspora delbrueckii* is characterized by lower production of acetic acid and ethanol, and higher yields of glycerol, fruit esters, thiols, and terpenes. It also positively contributes to the concentration of phenolic compounds and mannoproteins in wine, leading to increased levels of ethyl lactate, ethyl acetate, and 2-phenylethyl acetate [65,66]. The reduction in ethanol and acetic acid may result from the yeast’s ability to divert carbon flux from their synthesis toward the production of higher alcohols and esters via the α-ketoglutarate pathway, or toward glycerol synthesis through the glycerol-pyruvate pathway [67]. Compared to *S. cerevisiae*, *Schizosaccharomyces pombe* reduces the malic acid content and sourness in cider [41]. *Kluyveromyces marxianus* has been shown to produce high levels of ethyl acetate [68]. In cider making, the esters produced by *K. marxianus* were mainly acetates (ethyl acetate, propyl acetate, and hexyl acetate). Among these, the yield of ethyl acetate was 8.7–12.0 times higher than that produced by *Saccharomyces cerevisiae* [56].

However, non-*Saccharomyces* yeasts are sensitive to ethanol, limiting their industrial use. Additionally, the low concentration of assimilable nitrogen in apple juice can be rapidly depleted by yeasts during the early stages of fermentation. Consequently, some non-*Saccharomyces* yeasts are unable to complete alcoholic fermentation independently [69]. In order to ensure reliable fermentation while also enhancing the sensory profile of the wine, researchers have adopted mixed fermentations using both non-*Saccharomyces* and *Saccharomyces cerevisiae* yeasts [70]. Wines produced by co-culture of *M. pulcherrima* and *S. cerevisiae* were found to contain elevated concentrations of higher alcohols and esters relative to those from single-strain fermentation [71]. Sequential inoculation of *S. bayanus* and *T. delbrueckii* led to a 53.3% increase in ester content (predominantly ethyl esters) and a 32.8% reduction in acid content compared to single-strain fermentation [72]. However, antagonistic interactions in direct co-culture between *S. cerevisiae* and non-*Saccharomyces* yeasts often result in limited survival of the non-*Saccharomyces* species. *S. cerevisiae* produces antimicrobial peptides (1–10 kDa) that may inhibit competitors [73]. Microbial interactions are species- and strain-dependent, influenced by nutrient competition, differential ethanol tolerance, and the production of inhibitory or signaling metabolites [13]. Sequential inoculation provides an effective strategy to optimize the synergistic effect between *S. cerevisiae* and non-*Saccharomyces* yeasts. Sequential inoculation allows non-*Saccharomyces* yeasts to establish metabolic activity before *S. cerevisiae* dominates ethanol production [74]. Yuening Li et al. evaluated sequential inoculation of *M. koreensis* with *S. cerevisiae*. The results showed that a 48-h delayed inoculation extended the survival of *M. koreensis* to 9 days, increased ethanol production, and imparted sensory attributes characterized by reduced acidity and astringency alongside enhanced umami and richness [58].

Malolactic fermentation (MLF) significantly influences the aromatic complexity of fruit wines by generating odor-active compounds and transforming volatile compounds and flavor precursors from both the fruit and yeast [75]. MLF decarboxylates the major acid, malic acid, into lactic acid. Quinic and shikimic acids are converted into phenolic or other compounds. An alternative pathway for lactate formation is the reduction in pyruvate by lactic acid bacteria [76]. Following MLF, the content of flavonoids increases notably [4]. Lactic acid bacteria (LAB) are the primary agents of MLF. They can enhance the mouthfeel and aroma of the wine and improve its microbial stability. LAB from various genera, including *Lactobacillus*, *Pediococcus*, and *Oenococcus*, participate in this secondary wine fermentation. Spontaneous MLF is often unpredictable. To control the process and prevent off-flavors, the use of MLF starter cultures has become common practice [77]. *Oenococcus oeni* is the predominant LAB conducting MLF in fruit wines [78]. Inoculation with *Oenococcus oeni* rapidly reduced malic and citric acid content in cider. It also increased levels of lactic acid, ethyl lactate, fatty acids, and terpenes, while leaving higher alcohols unchanged [54]. Traditionally, MLF is conducted sequentially after alcoholic fermentation. However, the ethanol and metabolites produced by yeast can inhibit the growth of LAB strains, leading to failed MLF initiation. Currently, strains of *Lactobacillus plantarum* (reclassified as *Lactiplantibacillus plantarum*) have demonstrated resistance mechanisms that enable survival and proliferation in wine. Consequently, they are considered suitable starter cultures for inducing MLF. *Lactobacillus plantarum* UNQLp 11 exhibited glucosidase activity, leading to a significant increase in neutral polysaccharide concentration after being inoculated into wine [79]. Co-fermentation of *Lactiplantibacillus plantarum* and *Saccharomyces cerevisiae* increased the contents of ethyl acetate and isoamyl alcohol in cider and significantly enhanced its antioxidant capacity [57].

### 3.3. Process Precision: Oxygen and Nutrients

Factors including fermentation oxygen and additives (nutrients) collectively alter the chemical profile of cider, ultimately shaping its sensory characteristics.

Oxygen plays a dual role in the winemaking and aging process, exerting both positive and negative effects on wine quality [80]. During fermentation, oxygen primarily influenced wine composition by modulating yeast activity. During aging, micro-oxygenation promotes a series of reactions including oxidation, esterification, polymerization, and condensation, which significantly enhance the wine’s complexity and depth [81]. Under low-lipid conditions, oxygen supplementation induced yeast biosynthesis of ergosterol and unsaturated fatty acids, promoting nitrogen assimilation [82]. Research indicated that nitrogen directly impacts fermentation kinetics by reactivating protein synthesis (notably sugar transporters), while oxygen primarily affects late-stage kinetics by synthesizing unsaturated fatty acids and sterols, thereby increasing yeast ethanol tolerance [83]. Excessive oxygen, however, leads to negative outcomes such as phenol oxidation, increased tannin astringency and dryness, loss of freshness, and aroma oxidation [84]. One common mitigation strategy is the use of sulfur dioxide (SO_2_), which serves a dual purpose: inhibiting enzymatic/non-enzymatic oxidation and limiting microbial growth (Antioxidant strategies and mechanisms of cider fermentation with ergothioneine- and glutathione-producing yeasts: an alternative to sulfur dioxide.). Adding SO_2_ at 50 mg/L can reduce polyphenol oxidase activity by 75–90%. Furthermore, SO_2_ limits oxidation by reducing reaction products back to their original forms [85]. Micro-oxygenation (MOX) is an innovative technique in winemaking that enhances wine quality by facilitating slow, controlled oxidation. By precisely regulating the rate and amount of oxygen transfer, MOX achieved effects similar to those of long-term traditional aging in a shorter timeframe. This technology offered high batch-to-batch consistency and reproducibility, ensuring stable and predictable wine quality [86]. A recent strategy to reduce fruit wine alcohol content involves using aerobic conditions to allow respiro-fermentative metabolism of sugar. Non-*Saccharomyces* strains are employed to overcome limitations imposed by the Crabtree-positive character of *S. cerevisiae* [87]. Sequential fermentations with three non-*Saccharomyces* strains under different aeration conditions showed ethanol reductions of 1.6%, 0.9%, and 1.0% for *M. pulcherrima*, *T. delbrueckii*, and *Zygosaccharomyces bailii*, respectively, compared to *S. cerevisiae* wine [67].

Nutritional deficiencies, particularly in nitrogen and vitamins, are associated with stuck or sluggish fermentations. Therefore, external nutrients are sometimes added to unfermented juice to avoid such problems. Assimilable nitrogen is crucial not only for yeast biomass but also for synthesizing aromatic compounds [88]. It exists primarily as inorganic nitrogen (ammonium ions) or organic nitrogen (free amino nitrogen) [89]. Insufficient levels can lead to slow or stuck fermentation and promote the formation of undesirable volatile hydrogen sulfide [90]. Supplementation with 60 mg N/L of diammonium phosphate significantly increased the content of volatile compounds in cider, especially ethyl esters of medium-chain fatty acids and fatty acids. Adding 150 mg N/L of amino acids notably raised the concentrations of higher alcohols and acetate esters [91]. Yeast derivatives are widely used in winemaking for fermentation management and wine stabilization. They provide assimilable nitrogen, stimulate the growth of yeast and lactic acid bacteria, prevent sluggish fermentation, and enhance colloidal stability through the action of yeast mannoproteins [92]. The International Organisation of Vine and Wine (OIV) permits the use of yeast protein extracts for wine clarification. This practice reduces turbidity, preserves color and structure, removes excess tannins, and improves filterability [17].

## 4. Conclusions and Prospects

This review systematically elucidates that the final flavor profile of cider are the direct result of a complex interaction among three primary factors: the raw materials, the fermenting microorganisms, and the process control. The apple cultivar forms the foundational basis for flavor, with its specific sugar-to-acid ratio and polyphenol profile. The starter cultures, particularly the strategic shift from pure *S. cerevisiae* fermentations to simultaneous or sequential inoculations involving diverse non-*Saccharomyces* yeasts, have become the central driver for shaping aromatic complexity. LAB-driven malolactic fermentation also plays a critical role in shaping cider flavor. These microbial communities synergistically regulate the synthesis and balance of key aroma compounds. Precise control over key parameters—including oxygen management and nutrient supply—is essential for ensuring smooth fermentation and enhancing sensory quality.

Future research perspectives on cider are structured around the three key factors discussed above. Regarding raw materials, although a wide range of apple cultivars is available, the most suitable varieties for cider making remain unclear. Future efforts should therefore focus on selecting specific cultivars tailored to different cider styles, and standardizing the processing protocols for apple raw materials. Concerning starter cultures, although the use of non-*Saccharomyces* yeasts is becoming a trend in cider fermentation, completing alcoholic fermentation alone under relatively anaerobic conditions remains a challenge, limiting their commercial application. Given their Crabtree-negative trait, the application of micro-oxygenation and nutrient supplementation to improve their fermentation kinetics may offer a promising direction. In addition, research on malolactic fermentation (MLF) deserves further exploration. Beyond *Oenococcus oeni* and *Lactiplantibacillus plantarum*, more lactic acid bacteria strains that are well adapted to the cider environment and capable of co-fermentation with yeasts should be screened. With respect to process control, in addition to modulating microbial metabolism, pre-fermentation treatments such as cold maceration and enzymolysis should be investigated to enhance the extraction of soluble components from apples, thereby improving the flavor and functional properties of cider.

## Figures and Tables

**Table 1:** The richness of the European cider market.

Region	Product Name	Production Style
France	Cidre (Bouché)	bitter-sweet cider apples
Germany	Apfelwein	sharp and medium sharp apples fermented mostly to dryness
Spain	Sidra	sweet apples and bittersweet apples
Belgium	Cidre	mixed apples, some added pears
United Kingdom and Ireland	Cider	bitter-sweet cider apples fermented to dryness and sweetened after fermentation
Scandinavia	Cider	apple and pear juice is fermented to dryness and sweetened after fermentation

Resource: European Cider and Fruit Wine Association (https://aicv.org/en).

**Table 2:** The dominant volatile compounds in Cider.

Compounds	Concentration (mg/L)	Odor Description	Threshold (mg/L)
Higher Alcohols			
2-Methyl-1-propanol	0.036–0.63	Nail polish, alcohol	1.2
1-Butanol	0.041–0.16	Medicine, fruity	150
3-Methyl-1-butanol	0.72–36.4	Whiskey, malt, burnt	30
Benzyl alcohol	0.040–0.19	Floral, fruity	200
2-Phenylethanol	0.048–62.5	Honey, rose	10
1-Hexanol	0.043–3.37	Flower, green	8
1-Octanol	0.003–0.016	Chemical, metal, burnt	0.9
Volatile Acids			
Acetic acid	0.1–1.2	Vinegar-like	0.7
2-Methylpropanoic acid	0.4–2.0	Butter, cheese, rancid	30
Hexanoic acid	0.009–6.78	Cheese, sweat	0.42
Decanoic acid	0.16–1.78	Fatty	1
Octanoic acid	0.018–10.02	Fatty, rancid	0.5
Esters			
Ethyl acetate	2.40–125.7	Fruity, pineapple	7.5
Isoamyl acetate	0.0073–9.21	Fruity, sweet, banana	0.03
Hexyl acetate	0.0024–2.081	Green, herbaceous, fruity, grape	1.5
Ethyl butyrate	0.01–1.8	Fruity, sweet	0.02
Ethyl hexanoate	0.143–70.35	Fruity, green apple, banana	0.05
Ethyl octanoate	0.0014–119.6	Apple	0.02
Ethyl decanoate	0.004–50.86	Soap, floral	0.2
Diethyl sebacate	0.011–1.30	Green, fruity	0.1
Ethyl dodecanoate	0.027–6.94	Sweet, floral	0.8
Aldehydes			
Nonanal	0.10–0.24	Fatty, fruity, floral	/
Acetaldehyde	0.04–0.06	Pungent, ether	0.5
2-methylbutanal	0.003–0.1	Fruity, green grass	0.016
Benzaldehyde	0.0011–1.21	Almond, burnt, sugar	2
Terpenes			
β-Damascenone	0.06–0.20	Floral	/
Geranyl acetone	0.0096–0.04	Green, fruity	/

References: [18,19,20,21,22,23].

**Table 3:** Starter cultures and fermentation conditions of ciders.

Strains	Fermentation Parameters	Reference
*Torulaspora delbruecki*, *Saccharomyces cerevisiae*	Estonian apple varieties, Sequential inoculation, 21 °C	[44]
*Saccharomyces uvarum* *Hanseniaspora osmophila* *Hanseniaspora uvarum* *Torulaspora delbruekii* *Starmerella bacillaris*	Golden Delicious, 20 °C	[20]
*Pichia kluyveri*, *Hanseniaspora uvarum* *Pichia kluyveri*, *Hanseniaspora vineae* *Pichia kluyveri*, *Torulaspora quercuum* *Hanseniaspora vineae*, *Torulaspora quercuum*	Fuji apple, Simultaneous inoculation, 18 °C	[25]
*Saccharomyces cerevisiae*, *Oenococcus oeni*	Red-fleshed apple, Sequential inoculation, 18–20 °C	[54]
*Saccharomyces cerevisiae*, *Lactobacillus plantarum*	Fuji, Sequential inoculation 25 °C (yeast), 39 °C (lactic acid bacteria)	[55]
*Kluyveromyces marxianus*	Fuji apple, 30 °C	[56]
*Saccharomyces cerevisiae*, *Lactobacillus plantarum*	Fuji apple, Simultaneous inoculation, 20 °C	[57]
*Metschnikowia koreensis*, *Saccharomyces cerevisiae*	Fuji apple, Sequential inoculation, 28 °C	[58]
*Pichia kluyveri*, *Torulaspora delbrueckii*	Fuji apple, Sequential inoculation, 18 °C	[13]

## Data Availability

No new data were created or analyzed in this study. Data sharing is not applicable to this article.
